# Factors Associated with Intention to Receive the TAK-003 Dengue Vaccine Among Adults in Endemic Regions of Peru: A Cross-Sectional Study

**DOI:** 10.3390/vaccines14100894

**Published:** 2026-10-13

**Authors:** Jose Armando Gonzales-Zamora, Julieta Maribel Araoz-Salinas, Carlos Quispe-Vicuña, Gabriel Aquino-Sandoval, Martin Ernesto Reategui-Garcia, Allison Nicole Ortiz-Pardo, Abraham De-Los-Rios-Pinto, Noelia Morocho-Alburqueque, Silvia Celeste Tipe-Gonzales, Wagner Rios-Garcia, Anderson Nelver Soriano-Moreno, Brando Ortiz-Saavedra, José Miguel Rios-Osorio, Ruth Ramon-Tapia, Jorge Alave

**Affiliations:** 1 Peruvian American Medical Society, Ann Arbor, MI 48104-1059, USA; julieta.araoz@unmsm.edu.pe (J.M.A.-S.); nicoleortiz749@gmail.com (A.N.O.-P.); 2 Grupo de Investigación Neurociencias, Metabolismo, Efectividad Clínica y Sanitaria (NEMECS) y Centro Cochrane, Universidad Científica del Sur, Lima 15067, Peru; vicunas998@gmail.com; 3 Facultad de Medicina Humana, Universidad Nacional de la Amazonía Peruana, Iquitos 16000, Peru; 20091c0329@unapiquitos.edu.pe (G.A.-S.); martin.reategui.031@unapiquitos.edu.pe (M.E.R.-G.); 4 Facultad de Medicina Humana, Universidad Nacional de San Antonio Abad del Cusco, Cusco 08003, Peru; 174482@unsaac.edu.pe; 5 Facultad de Ciencias de la Salud, Universidad Nacional de Piura, Castilla, Piura 20002, Peru; dmorochoal@egresados.unp.edu.pe; 6 Instituto de Medicina Tropical Alexander von Humboldt, Universidad Peruana Cayetano Heredia, Lima 15102, Peru; silvia.tipe@upch.pe; 7 Facultad de Medicina, Universidad Nacional San Luis Gonzaga, Ica 11001, Peru; wagner16rg@gmail.com; 8 Unidad de Investigación Clínica y Epidemiológica, Escuela de Medicina, Universidad Peruana Unión, Lima 15464, Peru; andersonsoriano@upeu.edu.pe (A.N.S.-M.); jorgealave@upeu.edu.pe (J.A.); 9 Cardiovascular Surgery Department, Instituto Nacional Cardiovascular Carlos Alberto Peschiera Carrillo, EsSalud, Lima 15072, Peru; brandortizaavedra@gmail.com; 10 Facultad de Medicina “Hipólito Unanue”, Universidad Nacional Federico Villarreal, Lima 15088, Peru; jmro.med@gmail.com; 11 NYC Health + Hospitals/Lincoln, Bronx, NY 10451, USA; ramontar@nychhc.org

**Keywords:** dengue fever, vaccine, perception, intention, Peru

## Abstract

Background: Dengue vaccination is a promising preventive strategy. Peru introduced the TAK-003 vaccine in November 2024 for children aged 10–16 years and expanded it in January 2026 to individuals aged 10–20 years and selected priority adult groups. This study evaluated perceptions and intention to receive the dengue vaccine among adults before this expansion. Methods: We conducted an analytical cross-sectional study among adults living in Peruvian regions with medium or high dengue incidence. Participants completed a web-based questionnaire between 22 March and 13 April 2025 that assessed their perceptions of dengue vaccination and intention to receive the vaccine. To determine the associated factors, we estimated crude Prevalence Ratios (cPR) and adjusted Prevalence Ratios (aPR) with 95% Confidence Intervals (95% CI) using crude and multivariable Poisson regression models with robust variance. Results: We included 459 participants with a median age of 28 years; of them, 61.2% were women, and 73.6% lived in the coastal region. Overall, 91.5% intended to receive the dengue vaccine. Most considered the vaccine necessary (80.2%) and believed that it protected against severe dengue (67.1%). However, only 24.2% considered the information provided by the Peruvian Ministry of Health adequate. In the crude analysis, vaccination intention was associated with previous receipt of dengue vaccine information and several perceptions regarding vaccine necessity, effectiveness, adverse effects, and healthcare professional recommendations. After multivariable adjustment, only prior receipt of dengue vaccine information remained independently associated with greater vaccination intention (aPR: 1.05; 95% CI: 1.00–1.10; *p* = 0.039). Conclusions: Most participants intended to receive the dengue vaccine and generally reported favorable perceptions toward vaccination. Previous receipt of dengue vaccine information was independently associated with greater vaccination intention. Therefore, clear and evidence-based educational strategies may support informed vaccination decisions. Further research is needed to validate these findings and explore other factors associated with dengue vaccination.

## 1. Introduction

Dengue is a growing public health threat in tropical and subtropical regions. In 2024, the World Health Organization (WHO) reported 14,434,584 cases and 11,201 deaths, the highest global burden recorded to date [1]. Peru has experienced recurrent epidemics, particularly along the northern coast and in the Amazon region [1,2]. More recently, provisional surveillance data from the Peruvian Center for Disease Control and Prevention indicated that 46,669 dengue cases and 57 deaths had been reported by epidemiological week 33 of 2026, compared with 30,587 cases and 49 deaths during the same period in 2025, representing increases of 53% and 16%, respectively [3]. Although prevention has traditionally relied on surveillance, vector control, environmental management, personal protection, and timely diagnosis, maintaining these interventions at the required intensity is challenging during periods of widespread transmission. Vaccination has therefore emerged as an important complementary tool within an integrated dengue prevention and control strategy [4].

Dengue vaccine development has been challenging because protection must be balanced across the four viral serotypes and previous infections influence the risk of subsequent disease. CYD-TDV (Dengvaxia), the first licensed dengue vaccine, was restricted to individuals with evidence of previous dengue infection after an increased risk of hospitalization and severe dengue was identified among recipients who were seronegative before vaccination [4,5]. TAK-003 (Qdenga; Takeda), the second licensed dengue vaccine, is a live attenuated tetravalent vaccine that can be administered without pre-vaccination serological testing and is given as two-dose series three months apart [4]. In the phase 3 TIDES trial, conducted among 20,071 children and adolescents aged 4–16 years, TAK-003 showed 80.2% efficacy against virologically confirmed dengue and 95.4% efficacy against hospitalization [6]. However, direct evidence of clinical efficacy in adults remains limited [6,7].

The WHO recommends that countries consider introducing TAK-003 in settings with high dengue transmission and a substantial disease burden, primarily among children and adolescents aged 6–16 years [4]. The WHO does not currently recommend universal vaccination of adults; however, adults up to 60 years of age with comorbidities could be offered TAK-003 in endemic settings where there is a substantial risk of severe dengue. The vaccine is not recommended during pregnancy or breastfeeding or for individuals with congenital or acquired immunodeficiency [4].

The implementation of TAK-003 in Latin America has been heterogeneous. Brazil began public vaccination in February 2024 among children and adolescents aged 10–14 years living in prioritized municipalities and expanded the strategy to all municipalities in 2026 [8,9]. Argentina launched a targeted strategy in September 2024 for individuals aged 15–39 years living in high-risk departments [10]. In 2026, Paraguay expanded its program to people aged 6–39 years in prioritized municipalities [11]. These experiences show that access to vaccination is increasing across the region, although eligibility and coverage remain targeted rather than universal.

In Peru, the Ministry of Health (MINSA) launched a TAK-003 pilot vaccination program on November 23, 2024, in prioritized districts of Tumbes, Piura, Loreto, and Ucayali. The program targeted adolescents aged 10–16 years [12]. By the end of December 2025, the pilot program had achieved 100% first-dose coverage and 82% second-dose coverage among the target population [13]. In January 2026, National Health Technical Standard No. 243-MINSA/DGIESP-2026 expanded eligibility in prioritized regions to individuals aged 10–20 years residing in risk areas, healthcare professionals up to 59 years of age working in areas with a risk of dengue exposure, and individuals up to 59 years of age performing military service in such areas [14,15]. Thus, although selected groups of adults can currently access publicly funded vaccination, the program has not been extended to the general adult population.

The success of any future expansion of vaccination will depend not only on vaccine availability but also on public acceptance and trust. A meta-analysis estimated global dengue vaccine acceptance at 88.3%, although substantial differences were identified across populations and settings [16]. In Peru, a study conducted before vaccine availability in Piura and Iquitos found that, among 883 participants, 81.9% would accept vaccination, 14.5% were unsure, and 3.6% would refuse [17].

Despite these findings, evidence on dengue vaccination intention among adults across Peru’s endemic regions remains limited. Before further expansion to broader segments of the adult population, it is necessary to identify public doubts, fears, misconceptions, perceived need for vaccination, and trusted sources of information. This would help identify potential intervention targets and inform communication and implementation strategies that promote vaccine acceptance and completion of the two-dose series. Therefore, this study aimed to evaluate perceptions of dengue vaccination and identify factors associated with the intention to receive the TAK-003 vaccine among adults living in regions of Peru with medium and high dengue incidence.

## 2. Materials and Methods

### 2.1. Study Design, Setting, and Participants

**Figure 1:**
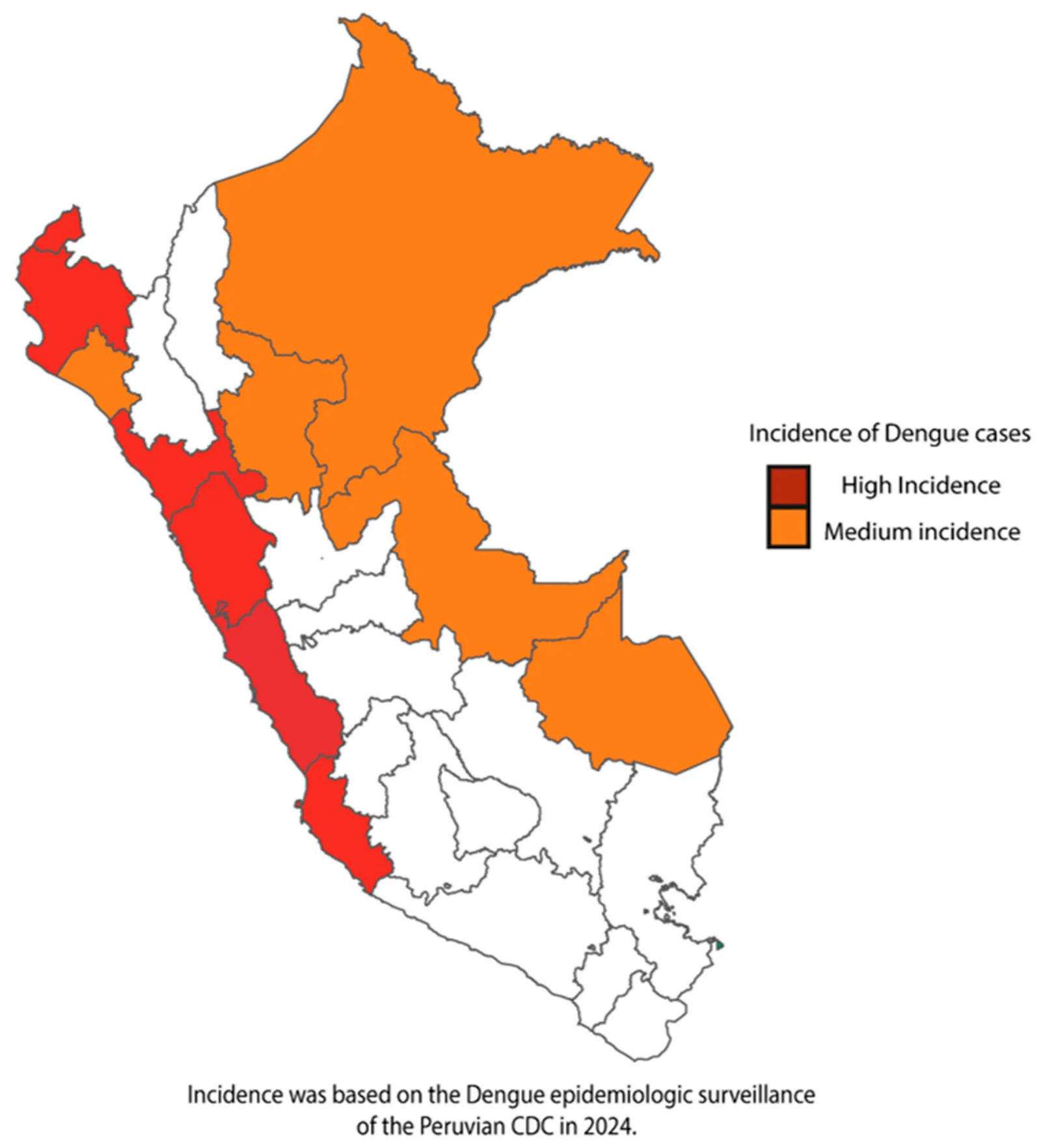
Peruvian regions with medium and high dengue incidence included in the study. Prepared by the authors using 2024 Peruvian CDC data [3].

We conducted an analytical cross-sectional study based on an online survey from 22 March to 13 April 2025. We collected data during a period when publicly funded vaccination in Peru remained restricted to the pediatric population through a national pilot program. We followed the STROBE guidelines for cross-sectional studies (Supplementary Material File S1) [18]. Peru is located on the western coast of South America, bordering the Pacific Ocean. The study population consisted of Peruvian adults aged ≥18 years residing in regions with medium or high dengue incidence: Ancash, Callao, Ica, La Libertad, Lambayeque, Lima, Loreto, Madre de Dios, Piura, San Martín, Tumbes, and Ucayali (Figure 1). We excluded adults who had received the dengue vaccine or had participated in clinical trials or studies on dengue vaccines in the last year.

### 2.2. Sample and Diffusion

We used the EpiData V4.2 program to calculate the sample size. Based on a population estimate of 22,038,203 inhabitants [19] for regions with moderate and high dengue incidence, as reported by the National Institute of Statistics and Informatics (INEI) in 2023, and an expected proportion of positive attitudes of 50%, the calculation yielded a minimum sample size of 385 participants, considering a 95% confidence level and a 5% margin of error. To account for incomplete responses or participants who did not meet the eligibility criteria, we increased the sample by 10%, resulting in a target of at least 424 participants. We used nonprobability snowball sampling to recruit participants, and we distributed the questionnaire through social media platforms: Facebook, Twitter, Instagram, and WhatsApp.

### 2.3. Questionnaire

Data were collected using an electronic questionnaire that was created in Google Forms (Supplementary Material File S2) and had 30 items organized into the following sections: (1) Inclusion criteria, (2) Exclusion criteria, (3) Sociodemographic data, (4) History of dengue infection, (5) Vaccination history, (6) Information about the dengue vaccine, (7) Perceptions of the risk of dengue infection, (8) Perceptions of the dengue vaccine, and (9) Intention to receive the dengue vaccine. This questionnaire was based on similar surveys used in previous studies conducted in Latin America (Argentina, Brazil, Colombia, Mexico, Venezuela) and Asia (Singapore, Malaysia, Indonesia) [20,21], as well as in Puerto Rico [22]. We adapted the questions to the Peruvian context and administered the questionnaire in Spanish. A panel of Peruvian infectious disease experts assessed content validity by evaluating each item for relevance, coherence, and clarity using a four-point scale ranging from 0 to 3. We considered their comments when preparing the final version of the questionnaire. A separate pilot test was not conducted because the items were adapted from previously used questionnaires and underwent expert content review.

### 2.4. Independent Variables

We collected sociodemographic characteristics, including age, sex, department and natural region of residence, healthcare worker status, educational attainment, and comorbidities. Dengue-related history included previous dengue infection, dengue infection during the preceding 12 months, hospitalization due to dengue, and dengue infection among first- or second-degree relatives. We also assessed previous receipt of other vaccines, previous receipt of information about the dengue vaccine, and the sources of this information.

Participants’ perceptions included the likelihood of acquiring dengue and of a family member becoming infected; the necessity of the dengue vaccine; its protection against mild dengue, severe dengue, and dengue-related mortality; concerns about serious adverse events and long-term negative effects; the importance of healthcare professional recommendations; and the adequacy of information provided by the Peruvian Ministry of Health. Participants were also asked whether they had children aged 10–16 years, whether their children had received the dengue vaccine, and whether they intended to vaccinate their unvaccinated children.

### 2.5. Dependent Variable

The dependent variable was intention to receive the dengue vaccine, assessed using the question: “Do you intend to receive the dengue vaccine when it becomes available?” The five response options were as follows: 1. “I will definitely get vaccinated,” 2. “I will probably get vaccinated,” 3. “I will probably not get vaccinated,” 4. “I will not get vaccinated,” and 5. “I have not decided yet.” Respondents who selected options 1 or 2 were classified as having the intention to receive it. Whereas those who selected options 3, 4, or 5 were classified as having no intention of receiving it.

### 2.6. Statistical Analysis

Statistical analyses were performed using R version 4 (R Foundation for Statistical Computing, Vienna, Austria). Categorical variables were summarized as absolute and relative frequencies, and numerical variables as means with standard deviations (SD) or medians with interquartile ranges (IQR), according to their distribution. Bivariate comparisons were performed using Fisher’s exact test for categorical variables and the Wilcoxon rank-sum (Mann–Whitney U) test for numerical variables. Factors associated with the intention to receive dengue vaccination were assessed by estimating crude (cPR) and adjusted Prevalence Ratios (aPR) with 95% Confidence Intervals (95% CI), using Poisson regression with robust variance. Variables with a *p*-value <0.10 in the bivariate analysis were included in the adjusted models. Statistical significance was defined as a *p*-value < 0.05.

### 2.7. Ethical Aspects

The study followed ethical standards and received prior approval from the Research Ethics Committee of Universidad Peruana Unión (approval number 2025-CEUPeU-002). Participants were informed about the study and provided informed consent before accessing the questionnaire. No personal identifying information was collected, and all study data were handled confidentially.

## 3. Results

Of the 510 survey respondents, 51 did not meet the eligibility criteria, resulting in a final sample of 459 adults. The median age was 28 years (IQR: 16), and most participants were women (61.2%), resided in the coastal region (73.6%), had completed higher education (89.1%), and were not healthcare workers (58.6%). Regarding clinical characteristics, 30.5% reported at least one comorbidity, 31.4% had a prior dengue diagnosis, and 50.1% had a first- or second-degree relative who had experienced dengue. Among participants with a previous dengue diagnosis (*n* = 144), 13.9% reported having had dengue within the previous 12 months and 22.9% reported a previous hospitalization due to dengue. Only 23.5% had previously received information about the dengue vaccine (Table 1).

**Figure 2:**
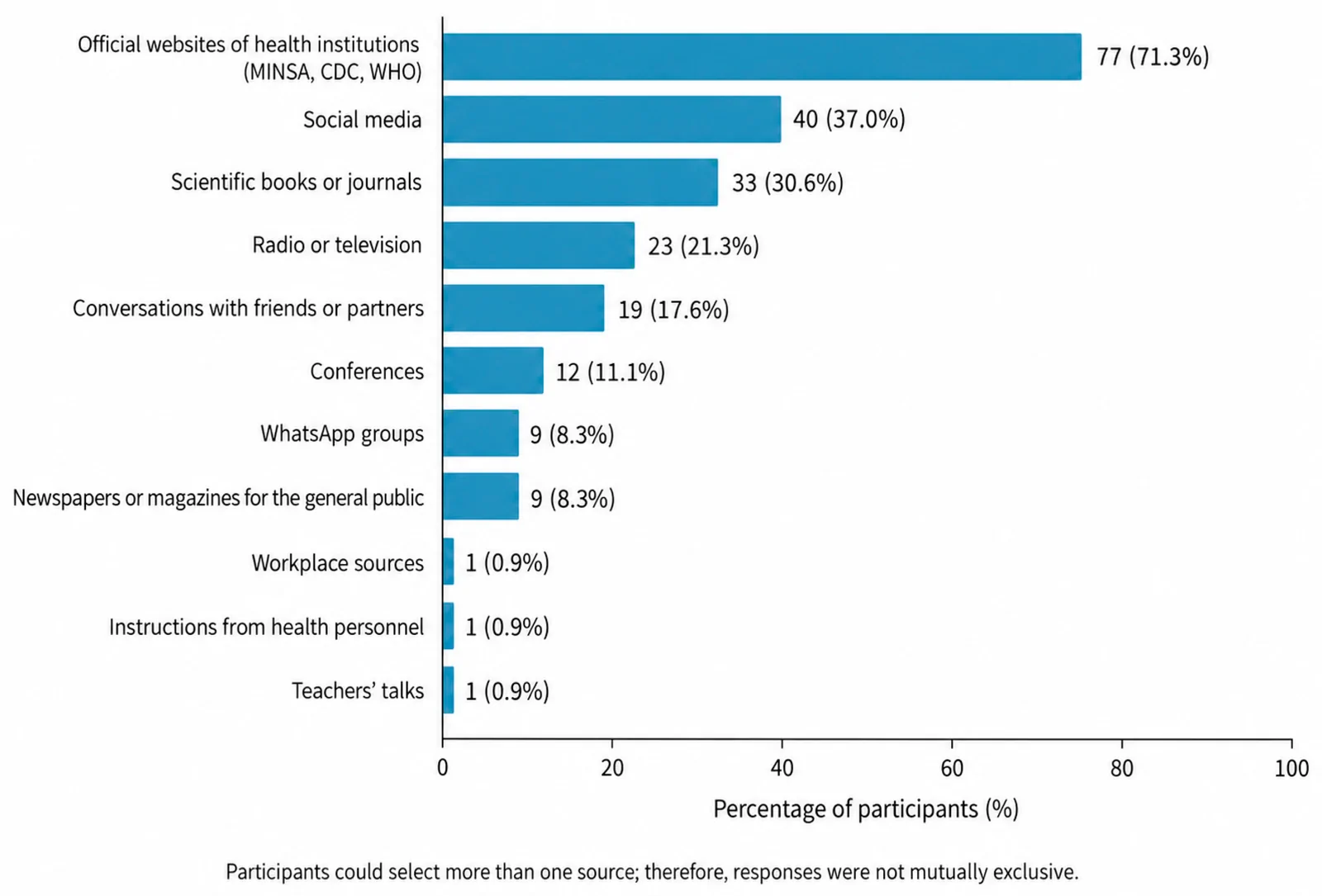
Sources of information about dengue vaccination among participants (*n* = 108).

Among the 108 participants (23.5%) who had received information about the dengue vaccine, official websites of health institutions, such as MINSA, the CDC, and the WHO, were the most frequently reported sources (77; 71.3%), followed by social media (40; 37.0%) and scientific books or journals (33; 30.6%). Since participants could select more than one source, the responses were not mutually exclusive. Radio or television was reported by 23 participants (21.3%), while 19 (17.6%) obtained information through conversations with friends or partners. Conferences were reported by 12 participants (11.1%), whereas WhatsApp groups and newspapers or magazines for the general public were each reported by 9 participants (8.3%). Workplace sources, guidance from healthcare professionals, and talks by teachers were each reported by 1 participant (0.9%) (Figure 2).

Regarding vaccination history, 98.7% of participants had received at least one of the vaccine types evaluated. The most frequent vaccination patterns involved five vaccines (26.4%), followed by six vaccines (22.9%) and four vaccines (19.8%). 10.9% of participants each reported receiving two or three vaccines, while 7.8% had received only one and 1.3% had received none. Among those who had received five vaccines, the most frequent combination included Coronavirus disease 2019 (COVID-19), hepatitis B, influenza, tetanus/diphtheria/pertussis, and yellow fever vaccines.

### 3.1. Perceptions of the Dengue Vaccine

More than half of the participants considered themselves likely to become infected with dengue (53.8%) and believed that a family member could become infected in the near future (57.5%). Most agreed that the dengue vaccine was necessary (80.2%) and protected against severe dengue (67.1%), mild dengue (60.8%), and dengue-related mortality (59.5%). In contrast, 13.1% believed that the vaccine frequently caused severe or serious adverse events, and 7.4% believed that it frequently caused long-term adverse effects. Recommendations from healthcare professionals were considered important by 90.0% of participants, whereas only 24.2% agreed that the Peruvian Ministry of Health provided adequate information about dengue vaccination (Table 2).

**Figure 3:**
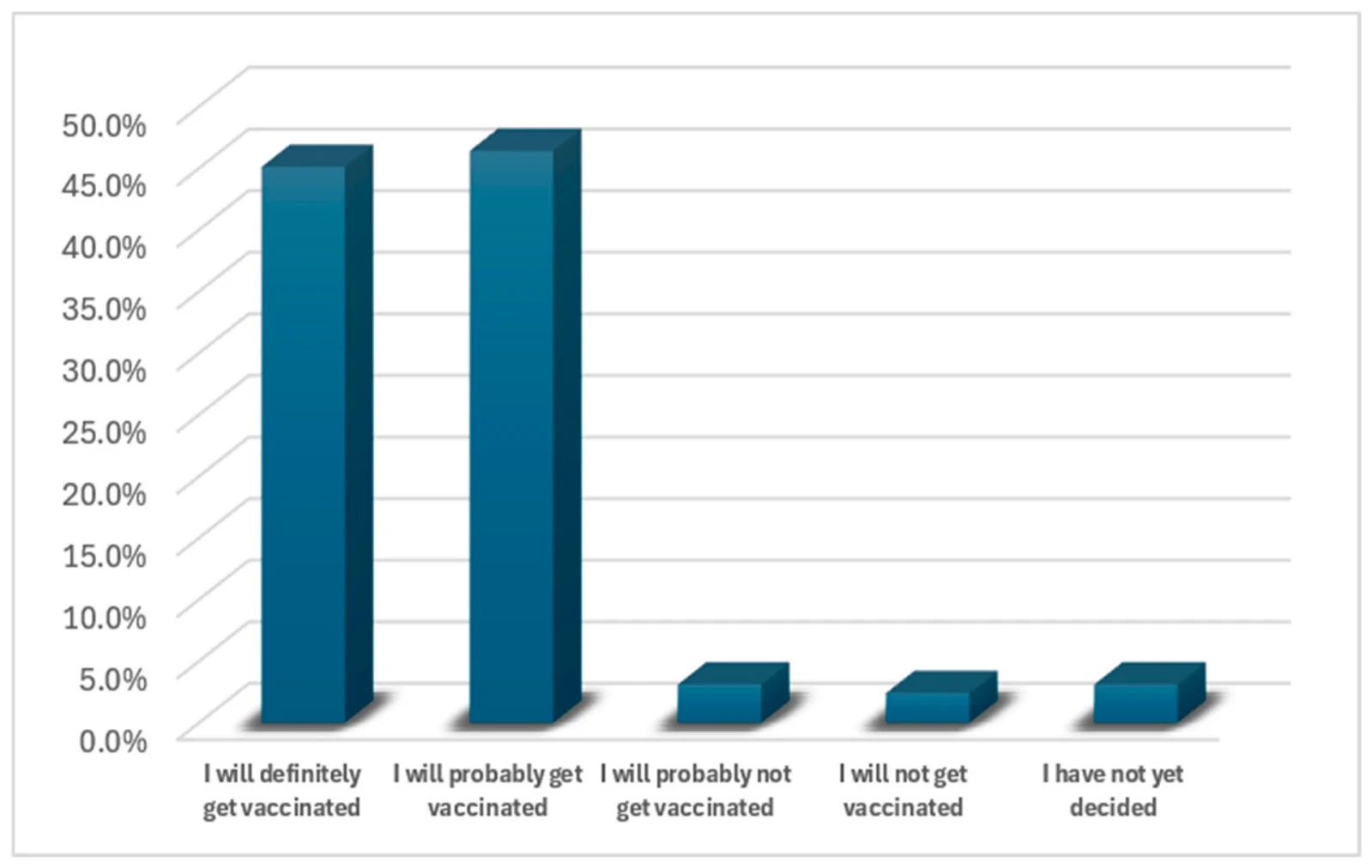
Intention of adults living in Peruvian regions with medium and high dengue incidence to receive the dengue vaccine.

We assessed the intention to receive the dengue vaccine using five response options. Of the 459 participants, 207 (45.1%) reported that they would definitely receive the vaccine, 213 (46.4%) would probably receive it, 14 (3.1%) would probably not receive it, 11 (2.4%) would not receive it, and 14 (3.1%) had not yet decided. For the analysis, the first two responses were grouped as “intention to receive the vaccine,” while the remaining three were grouped as “no intention or undecided.” Accordingly, 420 participants (91.5%) intended to receive the dengue vaccine, while 39 (8.5%) had no intention or were undecided (Figure 3).

Of the evaluated sample, 10.68% were parents of children aged 10–16 years. Among these parents, only 6.12% reported that their children had received the dengue vaccine. Regarding attitudes toward dengue vaccination, 14.29% (*n* = 7) had not yet decided whether to vaccinate their children, 4.08% (*n* = 2) were unlikely to vaccinate them, 42.86% (*n* = 21) were likely to vaccinate them, 32.65% (*n* = 16) stated that they would vaccinate their children regardless, and 6.12% (*n* = 3) indicated that they would not vaccinate them.

### 3.2. Bivariate Analysis

In the crude analysis, participants who received information about the dengue vaccine were more likely to report an intention to receive it than those who did not (cPR: 1.08; 95% CI: 1.03–1.14; *p* = 0.001). No meaningful association was observed between age and intention to receive the dengue vaccine (cPR: 1.00; 95% CI: 0.99–1.00; *p* = 0.038). Greater vaccination intention was also associated with considering the vaccine necessary (cPR: 1.45; 95% CI: 1.11–1.89; *p* = 0.007) and considering healthcare professionals’ recommendations important (cPR: 7.61; 95% CI: 1.22–47.62; *p* = 0.030). Conversely, lower vaccination intention was observed among participants who were uncertain whether the vaccine protects against dengue mortality (cPR: 0.86; 95% CI: 0.77–0.95; *p* = 0.004) and among those who believed that the vaccine frequently produces severe adverse events (cPR: 0.92; 95% CI: 0.87–0.98; *p* = 0.009) or long-term adverse effects (cPR: 0.82; 95% CI: 0.69–0.97; *p* = 0.023) (Table 3).

### 3.3. Multivariate Analysis

After adjustment, receiving information about the dengue vaccine remained significantly associated with vaccination intention across all three models. In Model 1, participants who had received vaccine-related information had a higher prevalence of vaccination intention (aPR: 1.08; 95% CI: 1.03–1.13; *p* = 0.002). This association remained significant in Model 2 (aPR: 1.07; 95% CI: 1.02–1.12; *p* = 0.005) and Model 3 (aPR: 1.05; 95% CI: 1.00–1.10; *p* = 0.039), although the magnitude of the association decreased slightly with additional adjustment. Age was significantly associated with vaccination intention only in Model 1 (aPR: 1.00; 95% CI: 0.99–1.00; *p* = 0.047), but this association was no longer statistically significant in Models 2 or 3. The perception that the dengue vaccine was necessary also lost statistical significance after adjustment, with an aPR of 1.22 (95% CI: 0.99–1.50; *p* = 0.067) in Model 1. In the fully adjusted Model 3, considering healthcare professionals’ recommendation of the dengue vaccine as important showed a borderline association with vaccination intention (aPR: 6.13; 95% CI: 1.00–37.73; *p* = 0.050). Other vaccine-related perceptions were not independently associated with vaccination intention in the adjusted models. We present in Table 3 only the results of model 3, which includes the variables from model 1 (general characteristics), model 2 (model 1 + probability of household infection) and the remaining variables with a *p*-value < 0.10 in the crude model (Table 3). No evidence of severe multicollinearity was identified in the fully adjusted model; the mean VIF was 2.48.

## 4. Discussion

In the present study, 91.5% of participants expressed intention to receive the TAK-003 dengue vaccine, indicating strong intention among adults living in dengue-endemic regions of Peru. Similar findings have been reported in Brazil, where vaccine acceptance was 93.6% [23], and Puerto Rico, where dengue vaccination intention was as high as 84.5% [22]. Lower rates have been found mainly in Southeast Asia, where studies in Thailand and Indonesia reported intentions of 68.7% and 51.0%, respectively [24,25]. A prior study in Peru found vaccine acceptance among adults to be 81.9%; however, it was limited to two endemic areas (Piura and Iquitos) and evaluated intention to receive a hypothetical future dengue vaccine [17]. Of note, our study aimed to assess adults’ intention to receive TAK-003, a vaccine that, during the data collection period, was available in Peru only to children and adolescents aged 10–16 years [26].

In 2026, Peru expanded its dengue vaccination program to individuals aged 10–20 years and frontline personnel, including healthcare workers, members of the Armed Forces, and police officers up to age 59. Still, it has not yet extended it to the general adult population [27]. Peru has not yet reported national coverage estimates for adults; however, by February 2026, Piura had vaccinated 7130 individuals from prioritized occupational groups in Piura, a northern region of Peru. This figure reflects initial uptake rather than coverage because the eligible population size and completion of the two-dose schedule were not reported [28]. Experiences from Brazil [29] and Argentina [30] illustrate the challenges of translating acceptance into vaccine uptake: Brazil reported approximately 4.8 million first doses but only 2.5 million second doses during 2024–2025, while Argentina distributed 170,000 doses to 11 provinces in 2026, sufficient for approximately 85,000 complete two-dose schedules. Therefore, the high intention observed in our study should be interpreted as a favorable indicator of potential vaccination intention rather than an estimate of future vaccination coverage.

Among the population characteristics observed in our study, we found that previous vaccination was highly prevalent, particularly against COVID-19, influenza, and hepatitis B. Other studies have also reported vaccination background as a factor associated with dengue vaccination intention. In Puerto Rico, participants who had received the influenza vaccine during the previous year showed greater dengue vaccination intention [22]. In Indonesia, willingness to receive a dengue vaccine was higher among participants vaccinated against COVID-19 than among those who were not vaccinated (53% vs. 15%) [25]. Although we did not analyze the association with vaccination background, the evidence suggests that prior vaccination behavior may reflect greater familiarity with and confidence in vaccines and should be investigated as a potential determinant of dengue vaccination intention in future Peruvian studies.

One of the main findings of our study was that prior receipt of information about the dengue vaccine was independently associated with a higher intention to receive it (aPR = 1.05; 95% CI: 1.00–1.10; *p* = 0.039). This association remained statistically significant across the three sequential multivariable models, although its magnitude was slightly attenuated after progressive adjustment (aPRs: 1.08, 1.07, and 1.05). Although the magnitude of this association was modest, this finding is relevant because access to information represents a potentially modifiable factor through health communication and educational interventions. However, the association was small, and its public health relevance remains uncertain. Given the cross-sectional, observational design, this association should not be interpreted as causal. This finding warrants further investigation because more than half of the participants in the present study perceived that the Ministry of Health was not providing adequate information about dengue vaccination. These results suggest a potential gap in institutional communication and support the need to strengthen information strategies with clear, evidence-based messages on vaccine safety, effectiveness, target populations, and vaccination schedules, particularly when delivered by healthcare professionals and trusted public health institutions [31].

Beyond the association between information exposure and dengue vaccination intention, our study provides insight into the channels through which participants received vaccine-related information. Among participants who reported information sources, official health institution websites, including MINSA, the CDC, and the WHO, were the most frequently reported (71.3%), followed by social media (37.0%) and scientific books or journals (30.6%). A similar pattern was observed in the multinational GEMKAP study, in which internet search engines (82%) and social media (62%) were the leading channels for obtaining health information across seven dengue-endemic countries [20]. In Pakistan, television was the most frequently used individual source of dengue-related information, followed by internet search engines, discussions with family and friends, and social media, based on a five-point frequency scale [32]. These results highlight the importance of digital channels as sources of dengue-related information. Therefore, official Peruvian websites and institutional social media accounts should provide clear, up-to-date, and evidence-based information about dengue vaccination.

Another important finding was the association between perceiving healthcare provider recommendations as important and intention to receive the dengue vaccine. Although this association did not reach statistical significance (aPR: 6.13; 95% CI: 1.00–37.73; *p* = 0.050), it was the largest association observed, with 90.0% of participants considering a professional recommendation important, although the marked imbalance across response categories limited the precision of its association with the vaccination decision. The wide confidence interval indicates substantial uncertainty in the magnitude of the association; nevertheless, the elevated point estimate suggests a potential relationship that warrants further evaluation in larger samples.

This pattern aligns with a broad body of evidence identifying HCP recommendation as one of the strongest drivers of vaccination intention across settings and vaccine types. A multi-country survey of HCPs in Latin America and Asia (including Argentina, Brazil, and Colombia) found that clinicians play an essential role in dengue vaccine uptake precisely because patients regard them as trusted sources of information [33]. A companion analysis of the same cohort, using a Capability–Opportunity–Motivation behavioral framework, also showed that HCPs’ knowledge, confidence, and motivation were the strongest predictors of their willingness to recommend the vaccine to patients [34], suggesting that strengthening provider-side confidence could lead to stronger patient-facing recommendations. In Peru, Cañari-Casaño et al. [17] also found that participants valued information from healthcare professionals and health institutions, particularly on vaccine safety, effectiveness, eligibility criteria, and potential adverse events. Studies evaluating other vaccines, such as COVID-19 vaccines, have similarly identified provider recommendation and trust in one’s personal doctor as among the strongest predictors of both intention and actual uptake, outweighing trust in religious leaders or family members [35]. When it comes to the HPV vaccine, receiving a recommendation from a provider has been associated with substantially higher odds of vaccination intention and initiation among parents, including in Latin American immigrant populations [36]. This overall effect likely reflects that a personalized, direct recommendation during a clinical encounter removes ambiguity and offers reassurance that generic public information campaigns cannot replicate [37].

These findings, together with ours, suggest that the role of HCPs in providing clear and reliable information about dengue vaccination warrants further consideration as the Peruvian vaccination program expands to the general adult population. Future communication strategies could train clinicians to routinely provide clear, evidence-based information about dengue vaccination during consultations, particularly given that clinician-related barriers, such as low confidence and motivation, have been shown to limit recommendation behavior even when knowledge is adequate [34]. Therefore, combining information from official digital platforms with brief guidance from trained healthcare professionals could help people access clear, reliable information about dengue vaccination.

Most participants (80.2%) considered dengue vaccination necessary, indicating a broadly favorable perception of vaccination. This perception was significantly associated with vaccination intention in the bivariate analysis (*p* = 0.007); however, the association was attenuated after adjustment and no longer reached statistical significance, although a positive trend remained (aPR = 1.22; 95% CI: 0.99–1.50; *p* = 0.067). This attenuation may reflect the influence of related factors, such as vaccine knowledge and access to information, which may partly account for the observed association. The high overall vaccination intention in our population (91.5%) may also have limited variability and reduced the ability to detect independent associations.

Several perceptions concerning vaccine benefits and safety were associated with dengue vaccination intention in the crude analysis. Although participants generally had favorable attitudes toward vaccination, substantial uncertainty remained regarding protection against mild and severe disease, dengue-related mortality, and possible long-term adverse effects. This coexistence of high intention and persistent uncertainty suggests that intention to receive the vaccine does not necessarily imply a complete understanding of the vaccine’s benefits and safety profile. Similar concerns have been documented in other endemic settings. In Brazil, negative perceptions of vaccine efficacy were associated with lower dengue vaccine acceptance [23]. At the same time, studies from Puerto Rico [38] and the Philippines [39] identified concerns about adverse events and insufficient safety information as important barriers to vaccination.

Available evidence regarding TAK-003 may help contextualize these concerns. During three years of follow-up in the phase 3 TIDES trial, the vaccine demonstrated sustained protection against symptomatic dengue and dengue-related hospitalization, with no major safety concerns identified [40]. Similarly, a Peruvian pharmacovigilance report covering the 2024–2025 vaccination pilot found that most reported events following immunization were mild or moderate, reports of serious events were uncommon, and nearly all affected individuals recovered completely [41]. Although pharmacovigilance reports do not establish that the vaccine caused the reported events, these findings provide locally relevant safety information. Communicating this evidence in clear, accessible language may help address misconceptions and support informed vaccination decisions.

The perceptions regarding safety and vaccine benefits did not remain independently associated with vaccination intention after multivariable adjustment. This may partly reflect the high prevalence of intention in our sample and the small number of participants classified as having no intention, which limited variability and may have reduced the precision of the estimates, particularly in the more extensively adjusted model. Therefore, the absence of independent associations should not be interpreted as evidence that perceptions of vaccine efficacy and safety are unimportant. Larger studies with more heterogeneous populations are needed to clarify their contribution to vaccination decisions. As adult vaccination strategies expand, Peruvian public health authorities should combine vaccine delivery with proactive communication through healthcare professionals, institutional digital platforms, and community-based activities. Experiences from Brazil [42] and Argentina [43] similarly emphasize the importance of addressing misinformation, clearly communicating vaccine benefits and limitations, and reinforcing the continued need for vector-control measures.

Future research should assess whether the high vaccination intention observed in this study translates into actual vaccine uptake and completion of the two-dose schedule as dengue vaccination is expanded to adult populations in Peru. Likewise, larger and more representative studies including participants from diverse geographic and sociodemographic settings are needed to further investigate the roles of previous vaccination history, access to reliable information, perceptions of vaccine safety and effectiveness, and trust in healthcare professionals and public health institutions. Finally, evaluating the effectiveness of specific communication and health education strategies could help identify interventions that promote informed decision-making and strengthen dengue vaccination programs.

### Limitations and Strengths

This study has several limitations. First, its cross-sectional design and online recruitment may have introduced selection bias and limited generalizability because participants were predominantly young, highly educated, and had internet access. Consequently, older adults, rural populations, and individuals with limited digital access may have been underrepresented, and the findings may not generalize to all Peruvian adults. Second, self-reported information may have introduced reporting and recall bias. In addition, during the data collection period, Peru restricted the dengue vaccine to children and adolescents aged 10–16 years. Therefore, adult participants did not have routine access to the vaccine, and their reported vaccination intention reflected a hypothetical scenario that may not translate into actual vaccine uptake once the vaccine becomes available to adults. Although we adapted the questionnaire to the Peruvian context, administered it in Spanish, and had a panel of Peruvian infectious disease experts assess content validity, we did not conduct a separate pilot test or formal psychometric validation; therefore, some measurement uncertainty may remain. Residual confounding from unmeasured or incompletely measured factors cannot be excluded despite adjustment for relevant covariates. Finally, the relatively small number of participants who reported no intention to receive the vaccine or remained undecided (*n* = 39) may have reduced statistical precision and contributed to unstable estimates and wide Confidence Intervals. Nevertheless, the study included 459 adults from multiple Peruvian regions with medium and high dengue incidence, providing evidence on dengue vaccination intention and its associated factors among adults in Peru and expanding the limited evidence available in this population. To our knowledge, this is the first study in the region to explore adults’ intention to receive the TAK-003 dengue vaccine.

## 5. Conclusions

The main finding of this study was that prior exposure to information about the dengue vaccine was independently associated with greater intention to receive it among adults living in regions of Peru with moderate and high dengue rates. However, the magnitude of this association was small, and its public health relevance remains uncertain. These findings support further evaluation of communication strategies that deliver clear, accurate, evidence-based information about dengue vaccination. Strengthening these strategies could foster greater intention to receive the vaccine and, potentially, improve vaccination intention and coverage as dengue vaccination expands to broader segments of the adult population. However, prospective studies involving more diverse populations are needed to assess whether intention to receive the vaccine translates into vaccination and adherence to the two-dose schedule.

## Figures and Tables

**Table 1:** Characteristics of the study population.

Characteristic	N = 459 (%)
Natural region	
Coast	338 (73.6%)
Jungle	121 (26.4%)
Age (years) *	28 (16)
Sex	
Female	281 (61.2%)
Male	178 (38.8%)
Healthcare worker	
No	269 (58.6%)
Yes	190 (41.4%)
Higher education	
No	50 (10.9%)
Yes	409 (89.1%)
Comorbidity	
No	319 (69.5%)
Yes	140 (30.5%)
Previous dengue infection	
No	315 (68.6%)
Yes	144 (31.4%)
Dengue infection within the last 12 months **	
No	124 (86.1%)
Yes	20 (13.9%)
Previous hospitalization due to dengue **	
No	111 (77.1%)
Yes	33 (22.9%)
Close family member (first- or second-degree relative) previously infected with dengue	
No	229 (49.9%)
Yes	230 (50.1%)
Received information about the dengue vaccine	
No	351 (76.5%)
Yes	108 (23.5%)

* median (IQR); ** Among participants who reported a previous dengue infection (*n* = 144).

**Table 2:** Perceptions regarding dengue and dengue vaccination.

Characteristic	N = 459 (%)
In your opinion, how likely are you to get dengue?	
Unlikely	182 (39.7%)
Not sure	30 (6.5%)
Likely	247 (53.8%)
In your opinion, how likely is it that a member of your family will get dengue soon?	
Unlikely	170 (37.0%)
Not sure	25 (5.4%)
Likely	264 (57.5%)
The dengue vaccine is necessary	
Disagree	27 (5.9%)
Neither agree nor disagree	64 (13.9%)
Agree	368 (80.2%)
The dengue vaccine protects against mild dengue	
Disagree	79 (17.2%)
Neither agree nor disagree	101 (22.0%)
Agree	279 (60.8%)
The dengue vaccine protects against severe dengue	
Disagree	33 (7.2%)
Neither agree nor disagree	118 (25.7%)
Agree	308 (67.1%)
The dengue vaccine protects against dengue-related mortality	
Disagree	71 (15.5%)
Neither agree nor disagree	115 (25.1%)
Agree	273 (59.5%)
The dengue vaccine frequently causes severe or serious adverse events	
Disagree	196 (42.7%)
Neither agree nor disagree	203 (44.2%)
Agree	60 (13.1%)
The dengue vaccine frequently causes long-term adverse effects	
Disagree	230 (50.1%)
Neither agree nor disagree	195 (42.5%)
Agree	34 (7.4%)
It is important to me that healthcare professionals recommend the dengue vaccine	
Disagree	8 (1.7%)
Neither agree nor disagree	38 (8.3%)
Agree	413 (90.0%)
The Peruvian Ministry of Health provides adequate information about dengue vaccination	
Disagree	237 (51.6%)
Neither agree nor disagree	111 (24.2%)
Agree	111 (24.2%)

**Table 3:** Factors associated with intention to receive the dengue vaccine.

Characteristics	Crude Analysis		Adjusted Analysis ^1^	
	cPR (95% CI)	*p*-Value	aPR (95% CI)	*p*-Value
Region				
Coast	Ref.			
Jungle	1.02 (0.96–1.08)	0.612		
Age (years)	1.00 (0.99–1.00)	0.038	1.00 (0.99–1.00)	0.219
Sex				
Female	Ref.			
Male	1.01 (0.96–1.07)	0.695		
Healthcare worker				
No	Ref.			
Yes	1.01 (0.96–1.07)	0.694		
Higher education				
No	Ref.			
Yes	0.99 (0.91–1.08)	0.891		
Presence of comorbidities				
No	Ref.			
Yes	1.02 (0.96–1.08)	0.471		
Previous dengue infection				
No	Ref.			
Yes	1.01 (0.96–1.07)	0.647		
Close relative (first- or second-degree) previously infected with dengue				
No	Ref.			
Yes	1.03 (0.98–1.09)	0.237		
Received information about the dengue vaccine				
No	Ref.		Ref.	
Yes	1.08 (1.03–1.14)	0.001	1.05 (1.00–1.10)	**0.039**
Perception of risk of dengue infection				
How likely do you think you are to get dengue?				
Unlikely	Ref.		Ref.	
Not sure	0.87 (0.72–1.04)	0.127		
Likely	1.00 (0.95–1.06)	1		
How likely do you think a member of your family will get dengue in the near future?				
Unlikely	Ref.		Ref.	
Not sure	0.82 (0.66–1.03)	0.089	0.85 (0.70–1.04)	0.125
Likely	1.00 (0.95–1.06)	0.978	1.01 (0.96–1.05)	0.761
Perceptions regarding the dengue vaccine				
The dengue vaccine is necessary				
Disagree	Ref.		Ref.	
Neither agree nor disagree	1.10 (0.81–1.49)	0.534	1.03 (0.81–1.32)	0.794
Agree	1.45 (1.11–1.89)	0.007	1.22 (0.99–1.50)	0.067
The dengue vaccine protects against mild dengue				
Disagree	Ref.		Ref.	
Neither agree nor disagree	0.99 (0.88–1.11)	0.813	1.03 (0.94–1.13)	0.585
Agree	1.08 (0.99–1.18)	0.076	1.04 (0.97–1.12)	0.230
The dengue vaccine protects against severe dengue				
Disagree	Ref.			
Neither agree nor disagree	1.04 (0.89–1.22)	0.639		
Agree	1.10 (0.95–1.28)	0.196		
The dengue vaccine protects against dengue-related mortality				
Disagree	Ref.		Ref.	
Neither agree nor disagree	0.86 (0.77–0.95)	0.004	0.94 (0.86–1.03)	0.209
Agree	1.01 (0.95–1.07)	0.774	1.01 (0.95–1.07)	0.675
The dengue vaccine frequently causes severe adverse events				
Disagree	Ref.		Ref.	
Neither agree nor disagree	0.92 (0.87–0.98)	0.009	1.01 (0.96–1.07)	0.734
Agree	0.94 (0.86–1.03)	0.203	1.04 (0.95–1.13)	0.400
The dengue vaccine frequently causes long-term adverse effects				
Disagree	Ref.		Ref.	
Neither agree nor disagree	0.90 (0.85–0.95)	<0.001	0.95 (0.90–1.00)	0.065
Agree	0.82 (0.69–0.97)	0.023	0.88 (0.76–1.02)	0.081
Recommendation of the dengue vaccine by healthcare professionals is important to me				
Disagree	Ref.		Ref.	
Neither agree nor disagree	5.47 (0.86–34.67)	0.071	4.97 (0.81–30.40)	0.083
Agree	7.61 (1.22–47.62)	0.03	6.13 (1.00–37.73)	0.050
The Peruvian Ministry of Health provides adequate information about dengue vaccination				
Disagree	Ref.			
Neither agree nor disagree	1.03 (0.97–1.10)	0.351		
Agree	1.04 (0.98–1.11)	0.206		

cPR: crude Prevalence Ratio; aPR: adjusted Prevalence Ratio; 95% CI: 95% Confidence Interval; Ref.: reference category. *p* values < 0.05 are shown in bold. ^1^ Adjusted by the variables with a *p*-value < 0.10 in the crude analysis.

## Data Availability

The authors will make the raw data supporting the conclusions of this article available upon request.

## Supplementary Materials

The following supporting information can be downloaded at https://www.mdpi.com/article/10.3390/vaccines14100894/s1. Supplementary Material File S1: STROBE Statement; Supplementary Material File S2: Survey.

## Abbreviations

The following abbreviations are used in this manuscript:

WHO	World Health Organization
MINSA	Ministry of Health
INEI	National Institute of Statistics and Informatics
HCP	Healthcare professional
