# Supplementary material for: Factors Associated with Intention to Receive the TAK-003 Dengue Vaccine Among Adults in Endemic Regions of Peru: A Cross-Sectional Study
Source: Vaccines (Basel). 2026 Oct 13;14(10):894. doi: 10.3390/vaccines14100894 (PMC13709946; doi:10.3390/vaccines14100894)
Supplement: Supplementary file 1 [file vaccines-14-00894-s001.zip › Supplemental material file S2. SURVEY.pdf]

Supplementary Material File S2: SURVEY

Exploring the intent to get vaccinated against dengue among adults from regions with medium and high dengue incidence in Peru

INCLUSION CRITERIA

1. In which department do you live?

   A. Piura
   B. Lambayeque
   C. La Libertad
   D. Ancash
   E. Lima
   F. Ica
   G. Loreto
   H. San Martín
   I. Ucayali
   J. Madre de Dios
   K. Tumbes
   L. Other (end the survey)

2. Are you an adult (18 years of age or older)?
   A. NO (end the survey)
   B. YES (proceed to question 3)

3. How old are you?
   Age in years: _____________ (18 or older)

EXCLUSION CRITERIA

4. Have you received the dengue vaccine?
   A. YES (end the survey)
   B. NO

5. Have you participated in any study or clinical trial on dengue vaccines in the past year?
   A. NO
   B. YES (end the survey)

SOCIODEMOGRAPHIC CHARACTERISTICS

6. Indicate your sex
   A. Male
   B. Female

7. Are you a healthcare worker?
   A. NO
   B. YES

8. What is your highest level of education?

A. Primary education
B. Secondary education
C. University / Higher education
D. Technical education
E. None of the above

9. Which health conditions do you have? You may select more than one option.
    A. Obesity
    B. Diabetes
    C. Cancer
    D. Kidney disease
    E. Heart disease
    F. Lung disease
    G. Nervous system disorder (stroke, seizures, etc.)
    H. High blood pressure
    I. Organ transplant
    J. Other, please specify: _____________
    K. None

HISTORY OF DENGUE

10. Have you ever had dengue?
    A. No (proceed to question 13)
    B. Yes (proceed to question 11)

11. Have you had dengue in the past 12 months?
    A. No
    B. Yes

12. Have you ever been hospitalized for dengue?
    A. No
    B. Yes

13. Has any close family member (a first- or second-degree relative) ever had dengue?
    A. No
    B. Yes

VACCINATION HISTORY

14. Which of the following vaccines have you received? Select all that apply.
    - o COVID-19 vaccine
    - o Hepatitis B vaccine
    - o Influenza vaccine
    - o Meningococcal vaccine
    - o Tetanus, diphtheria, and pertussis vaccine
    - o Yellow fever vaccine
    - o None of the above

INFORMATION ABOUT THE DENGUE VACCINE

15. Have you received information about the dengue vaccine?
    A. NO (proceed to question 17)

B. YES (proceed to question 16)

16. Through which sources have you obtained information about the dengue vaccine? (You may select more than one option.)
    - o I have not received any information
    - o Official websites of health institutions: MINSA, CDC, WHO, etc.
    - o Radio or television
    - o Newspapers or general-interest magazines
    - o Social media (Facebook, Instagram, Twitter, TikTok)
    - o WhatsApp message chains
    - o Conversations with friends or a partner
    - o Books or scientific journals
    - o Conferences
    - o Other sources, please specify: _____________

PERCEIVED RISK OF INFECTION

17. In your opinion, how likely are you to contract dengue?
    A. Very likely
    B. Likely
    C. Unlikely
    D. Very unlikely
    E. Not sure
18. In your opinion, how likely is your family to contract dengue in the near future?
    A. Very likely
    B. Likely
    C. Unlikely
    D. Very unlikely
    E. Not sure

PERCEPTIONS OF THE DENGUE VACCINE

Vaccines are substances that strengthen the immune system to help the human body defend itself against infections.

Since November of this year, the Ministry of Health has been administering the dengue vaccine free of charge to children aged 10 to 16. With regard to the DENGUE VACCINE, please answer the following questions:

19. What is your opinion of the following statement? The dengue vaccine is necessary.
    A. Strongly agree.
    B. Agree.
    C. Neither agree nor disagree.
    D. Disagree.
    E. Strongly disagree.

20. What is your opinion of the following statement? The dengue vaccine protects against mild cases of dengue.
    A. Strongly agree.
    B. Agree.
    C. Neither agree nor disagree.
    D. Disagree.

E. Strongly disagree.

21. What is your opinion of the following statement? The dengue vaccine protects against severe cases of dengue.
    A. Strongly agree.
    B. Agree.
    C. Neither agree nor disagree.
    D. Disagree.
    E. Strongly disagree.

22. What is your opinion of the following statement? The DENGUE vaccine protects against death from DENGUE.
    A. Strongly agree.
    B. Agree.
    C. Neither agree nor disagree.
    D. Disagree.
    E. Strongly disagree.

23. What is your opinion of the following statement? The dengue vaccine frequently causes severe or dangerous adverse effects.
    A. Strongly agree.
    B. Agree.
    C. Neither agree nor disagree.
    D. Disagree.
    E. Strongly disagree.

24. What is your opinion of the following statement? The dengue vaccine may frequently cause negative long-term effects.
    A. Strongly agree.
    B. Agree.
    C. Neither agree nor disagree.
    D. Disagree.
    E. Strongly disagree.

25. How IMPORTANT is it to you that a healthcare professional recommend the dengue vaccine?
    A. Very important
    B. Somewhat important
    C. Slightly important
    D. Not at all important
    E. Not sure

26. Do you agree with the following statement? The Peruvian Ministry of Health is providing adequate information about dengue vaccination.
    A. Strongly agree.
    B. Agree.
    C. Neither agree nor disagree.
    D. Disagree.

E. Strongly disagree.

INTENTION TO RECEIVE DENGUE VACCINATION

27. Do you intend to be vaccinated against dengue when the vaccine becomes available?
    - A. I will definitely get vaccinated
    - B. I will probably get vaccinated
    - C. I will probably not get vaccinated
    - D. I will not get vaccinated
    - E. I have not decided yet

28. Do you have children between 10 and 16 years of age?
    - A. No (END THE SURVEY)
    - B. YES (PROCEED TO QUESTION 29)

29. Did they receive the dengue vaccine?
    - A. YES (END THE SURVEY)
    - B. NO (PROCEED TO QUESTION 30)

30. Do you intend to have your child/children vaccinated?
    - A. I will definitely have them vaccinated
    - B. I will probably have them vaccinated
    - C. I will probably not have them vaccinated
    - D. I will not have them vaccinated
    - E. I have not decided yet
